# Design of a Depth Control Mechanism for an Anguilliform Swimming Robot

**DOI:** 10.3390/biomimetics6020039

**Published:** 2021-06-09

**Authors:** Ahmed Islam, Brandon Taravella

**Affiliations:** Department of Naval Architecture and Marine Engineering, University of New Orleans, New Orleans, LA 70148, USA; aislam@uno.edu

**Keywords:** anguilliform, biomimetics, swimming robot

## Abstract

This paper discusses the design and implementation of a depth control mechanism for an anguilliform swimming robot. Researchers analyzed three different methods of controlling the depth of the robot, including out-of-plane thrust direction, use of foil on the head and buoyancy control at the head and tail. It was determined that buoyancy control at the head and tail was the best method for controlling depth and pitch, given typical forward speeds of the robot. Details are given into the design of this mechanism, including a stress analysis on a critical part, as well as the impacts that these modifications have on the required torque of the drive servos.

## 1. Introduction

In recent years, the extensive need in underwater search, rescue, intelligence and combat operations has prompted researchers to develop aquatic robots that can survive in the environments of rivers and seas. Many researchers have focused on developing robotic fish of various types. These include robotic fishes whose swimming patterns classify under one of these four forms: carangiform, sub-carangiform, thunniform and anguilliform. These robotic fishes can be used as platforms for remotely controlled underwater vehicles, which are further known as autonomous underwater vehicles (AUVs) and sometimes as underwater drones and/or unmanned underwater vehicles (UUVs). 

A primary purpose of developing an underwater vehicle is to partake in what is known as ISR (intelligence, surveillance and reconnaissance) missions. One key characteristic of such missions is to carry out operations where significant acoustic, radar and optical signatures must be minimized. Under ideal fluid conditions, it has been long established that self-propulsion can occur [1,2], given that certain motion prerequisites are met. In an ideal fluid situation, it is thus expected to have very low aquatic signature, i.e., self-propulsion without wake and vortex trailing and minimally induced drag force. With early researchers proposing spatial wave propulsion models [3,4], later researchers have investigated various forms of locomotion using different mathematical and numerical models [5,6,7,8,9,10]. Idealized swimming reducing wake structure of an anguilliform-shaped body has therefore been proposed by Vorus [11] and Vorus and Taravella [12]. 

Even though conventionally designed AUVs and other underwater robots are typically equipped with traditional propeller type propulsion, there are certain drawbacks, including very high energy utilization and significant noise generation [13,14]. For this paper, we investigated underwater motion control of different types using multiple techniques to make a comparable study on the efficiency of depth control while an anguilliform AUV is swimming. Morgansen et al. [15] used open loop control for motion generation with fin actuation to direct the heaving and pitching of small 8.77 cm hydrofoils acting as pectoral fins. Zhang et al. [16] used pectoral fins made of organic glass controlled by a servomotor and connected by a rotating shaft. Low [17] experimented using a combination of undulating anal fins and a buoyancy control mechanism. While both the control systems are kept separate from each other, the buoyancy control consisted of proximity sensors, depth and pitch sensors, servo motors and microchip processing modules. Makrodimitris et al. [18] used a volumetric space control mechanism, as opposed to a fin-type control, where a small pump drives the volume of a bladder, also known as ballast tank. Makrodimitris et al. argued that the fin type approach is not practical due to the difficulty of operating at a very low or zero velocity. Sumantr et al. [19] proposed a simulation model to fill a water ballasting tank by the battery operating movement of a flat plate. Minh-Thuan et al. [20] experimented a lead screw system that can convert a pure rotation to a pure translation of a piston that is controlled by a servo motor via gearbox and transmission actuator. Inoue et al. [21] used the spermaceti oil hypothesis to implement a buoyancy control system that can heat and cool paraffin wax by the use of a Peltier element and a nichrome wire. Other notable buoyancy control systems for underwater robots include that of Detweiler et al. [22], who tested a depth and buoyancy engine of an underwater robot named AMOUR V, by moving a piston in a cylinder which was packaged as an auxiliary module. Many researchers chose to use bladder systems and buoyancy control systems over different types of fin systems due to space configuration, energy payload restrictions and other design limitations. 

In this paper, we present the design process of three depth control mechanisms to aid in smooth tri-directional swimming performance of the eel robot. These three methods of depth control include: (1) modification of servo orientation to redirect thrust, (2) addition of control surfaces to provide lift and sinkage and (3) control of displaced volume by having expandable/contractable components. The paper is organized as follows: First, we will present the theoretical background behind the ideal swimming of the anguilliform eel. This hydrodynamic theory is the methodology implemented to predict the inertial forces on the eel robot’s body, which are then used to predict the torque on the servo motors. Second, we will present the aspects of the design for the original robot that only undulates in the x–y plane. Finally, we will present the concept of depth change and modified fabrication and discuss the experimental results. 

## 2. Theoretical Background

**Figure 1:**
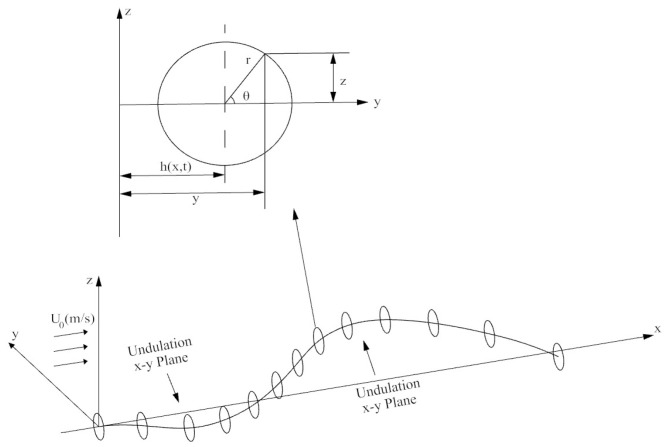
Analytical formulation of the anguilliform swimming motion in Cartesian and polar coordinates.

The basis of the theoretical formulation is presented in Vorus and Taravella [12]. A brief description is provided here for a basic understanding of how the hydrodynamic forces were computed for the current work. A basic schematic of the anguilliform swimmer is shown in Figure 1.

Slender body theory is implemented such that the sectional forces are computed for then being integrated along the length of the body to compute the total forces. A circular cross section at distance x from the origin is used to illustrate the cross-sectional surface of the anguilliform swimmer and defined by the following equation:

(1) $$F\left(x,y,z\right)=-y+h\left(x,t\right)+\sqrt{r_{0}^{2}-z^{2}}$$

where h(x,t) is the displacement of the undulated body in the x–y plane and r_0_ is the radius of the body.

Considering the velocity vector $\vec{V}=\left(U_{0}+u\right)\hat{i}+v\hat{j}+w\hat{k}$ where U_0_ is the free stream velocity and (u,v,w) is the perturbation velocity in (x,y,z), the derivative of F can produce the kinematic boundary condition for a cylindrical surface:

(2) $$h_{t}\left(x,t\right)+U_{0}h_{x}\left(x,t\right)=v,\;\mathrm{for}\;r=r_{0};\;0\leq x\leq L,$$

The ideal flow theory is used to compute the inertial forces. Justification for the use of the ideal flow theory is found in Vorus and Taravella [12]. For a 2D case, the perturbation velocity potential of a circular cross-section is given as:

(3) $$\phi \left(x,r,\theta ,t\right)=-\frac{v\left(x,t\right)r_{0}^{2}\cos\theta }{r},$$

In the 3D case, that is, the cylindrical perturbation, the following equation can be derived:

(4) $$\phi \left(x,\theta ,t\right)=-\left(h_{t}+U_{0}h_{x}\right)r_{0}\cos\theta$$

Utilizing Bernoulli’s equation, the linearized pressure is:

(5) $$p_{\mathrm{linear}}\left(x,\theta ,t\right)=-\rho \left(\phi_{t}+U_{0}\phi_{x}\right)$$

The unit pressure for the cylinder thus becomes:

(6) $$p\left(x,\theta ,t\right)=\rho \left(h_{\mathrm{tt}}+2U_{0}h_{\mathrm{xt}}+U_{0}^{2}h_{\mathrm{xx}}\right)r_{0}\cos\theta$$

By integrating the unit pressure over the circular cross section, the sectional forces per unit length can be written as:

(7) $$f_{x}\left(x,t\right)={\rho \pi r}_{0}^{2}\left(h_{\mathrm{tt}}+2U_{0}h_{\mathrm{xt}}+U_{0}^{2}h_{\mathrm{xx}}\right)h_{x}\left(x,t\right)$$

(8) $$f_{y}\left(x,t\right)=-{\rho \pi r}_{0}^{2}\left(h_{\mathrm{tt}}+2U_{0}h_{\mathrm{xt}}+U_{0}^{2}h_{\mathrm{xx}}\right)$$

in the x and y directions, respectively.

Vorus and Taravella [12] go on to develop an equation for undulatory motion that theoretically results in wakeless swimming by determining the shape function necessary to produce 100% Froude efficiency:

(9) $$\bar{h}\left(\bar{x},\bar{t}\right)=\;\Gamma \left[\sin\left(2\pi \left(\frac{\bar{x}}{U}-t\right)\right)-\sin\left(2\pi \left(\bar{x}-\bar{t}\right)\right)\right],$$

where Γ is the displacement amplitude that is based on desired thrust and the radius of the cross-section and $\bar{x}$ and $\bar{t}$ are the normalized position and the normalized time, respectively.

## 3. Design and Experimental Results

Before we discuss the anguilliform depth control design, we will present the NEELBOT without the depth control actuation that swims in undulatory form in a single x–y plane. The original anguilliform eel was 1.3 m long, with cylindrical body having a diameter of 0.055 m and a waterproof latex skin that was 0.5 mm thin. The forward and the aft ends were made of dome-shaped semi-spheres and the eel was measured to have a total mass of 3216.0 g. To achieve near perfect buoyancy while reducing chances of sinking and rolling, lead tape ballasting was used underneath the eel to lower the vertical center of gravity. The average experimented temperature for the water was measured at 16 °C and the density was 998.905 kg/m^3^. The servo actuators used in this study were the closed loop PID controlled Dongbu Herkulex DRS-0201 containing permanent magnet direct current (PMDC) motor. Each Dongbu Herkulex was powered by two sets of NiMH AAA batteries connected in parallel and controlled remotely using an onboard XBee wireless controller which fed relative motion and angular rotation data, that were programmed in LabVIEW. The key design parameters are shown in Table 1. 

**Figure 2:**
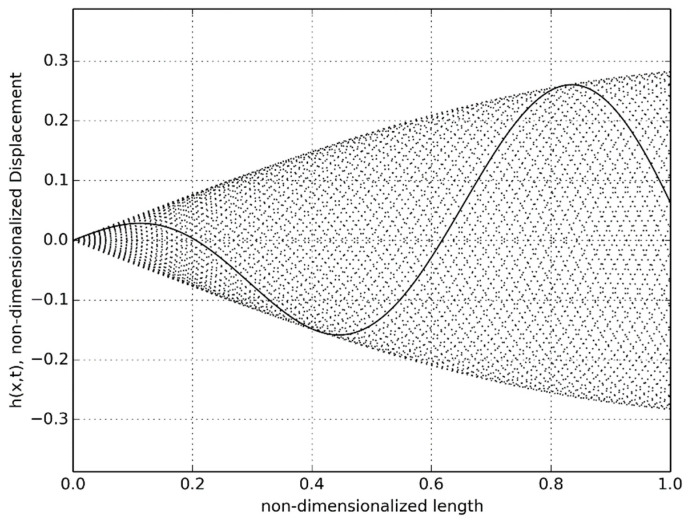
Nondimensionalized undulatory displacement over time.

**Figure 3:**
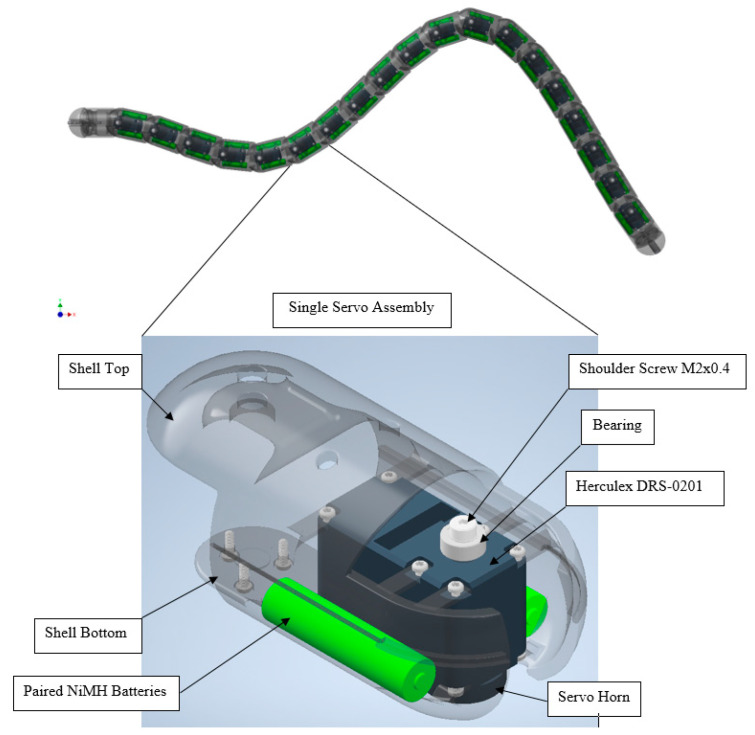
Shell assembly of the segment showing the servo motor, the shell, the batteries (wiring not shown) and other miscellaneous components.

Under ideal swimming motion, the predicted non-dimensionalized displacement of the anguilliform robot is shown in Figure 2. All the 19 segments with the installed Dongbu Herculex servos and the paired NiMH batteries did remain functioning during the feedback-controlled motion to produce the ideal undulatory motion. A detail of the servo and battery arrangement is shown in Figure 3.

### 3.1. Segment-Wise Nodal Force and Moment Decomposition

**Figure 4:**
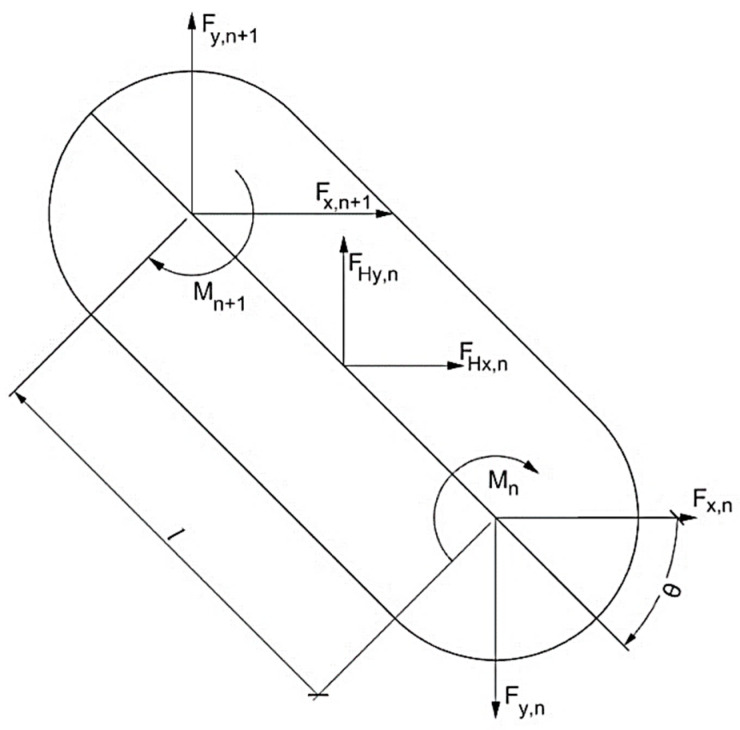
Segment free-body diagram.

In order to determine the required torque of the servo motors, it was necessary to compute the segment-wise forces and the moments at the joints in order to address the hydrodynamic forces acting on the robot [23]. A free-body diagram of an eel robot segment is shown in Figure 4.

The force decomposition showed that at the nodal points of the designed swimming segment n could be written as follows:

(10) $$\sum F_{x}=m_{n}A_{X},$$

(11) $$\sum F_{y}=m_{n}\left(a_{n,y}+A_{y}+\alpha_{E}l_{N}\left(-\frac{N}{2}+n-0.5\right)\right),$$

(12) $$\sum M_{z}=I_{n}\left(\alpha_{z,n}+\alpha_{E}\right),$$

where $m_{n}$ is the mass of segment. $a_{n}$ and $A_{x}$ are the local and global accelerations, respectively. $\alpha_{z}$ and $\alpha_{E}$ are the local angular acceleration around out-of-plane axis and global angular acceleration, respectively. $A_{y}$ and $a_{n,y}$ are the global acceleration and local acceleration in the y-direction at the midpoint of the segment. Equating the left side of the equation, the resulting force and moments could be written as follows:

(13) $$\sum F_{X}=F_{x,n}-F_{x,n+1}+F_{H_{x},n},$$

(14) $$\sum F_{y}=-F_{y,n}+F_{y,n+1}+F_{H_{y},n},$$

(15) $$\sum M_{z}=-M_{n}+M_{n+1}+\frac{\left(F_{y,n+1}+F_{y,n}\right)l_{n}}{2}\cos\theta -\frac{\left(F_{x,n+1}+F_{x,n}\right)l_{n}}{2}\sin\theta ,$$

Force-moment composition could be rearranged in terms of the following:

(16) $$A\underline{f}=B,$$

with

(17) $$A=\left[\begin{array}{cccccccccc} 1 & 0 & 0 & -1 & 0 & 0 & \cdots & -m_{n} & 0 & 0\\ 0 & -1 & 0 & 0 & 1 & 0 & \cdots & 0 & -m_{n} & -m_{n}l_{n}\left(-\frac{N}{2}+n-0.5\right)\\ -\frac{l_{n}}{2}\sin\theta & \frac{l_{n}}{2}\cos\theta & -1 & -\frac{l_{n}}{2}\sin\theta & -\frac{l_{n}}{2}\cos\theta & 1 & \cdots & 0 & 0 & -I_{n}\\ \vdots & \vdots & \vdots & \vdots & \vdots & \vdots & \ddots & \vdots & \vdots & \vdots \\ 1 & 0 & 0 & -1 & 0 & 0 & \cdots & -m_{n} & 0 & 0\\ 0 & -1 & 0 & 0 & 1 & 0 & \cdots & 0 & -m_{n} & -m_{n}l_{n}\left(-\frac{N}{2}+n-0.5\right)\\ -\frac{l_{n}}{2}\sin\theta & \frac{l_{n}}{2}\cos\theta & -1 & -\frac{l_{n}}{2}\sin\theta & -\frac{l_{n}}{2}\cos\theta & 1 & \cdots & 0 & 0 & -I_{n} \end{array}\right],$$

(18) $$\underline{f}=\left[\begin{array}{c} F_{x,n}\\ F_{y,n}\\ M_{n}\\ F_{x,n+1}\\ F_{y,n+1}\\ M_{n+1}\\ \vdots \\ A_{x}\\ A_{y}\\ \alpha_{E} \end{array}\right],$$

and

(19) $$B=\left[\begin{array}{c} -F_{H_{x},n}\\ m_{n}a_{n,y}-F_{H_{y},n}\\ I_{n}\alpha_{z,n}\\ \vdots \\ -F_{H_{x},N}\\ m_{N}a_{N,y}-F_{H_{y},N}\\ I_{N}\alpha_{z,N} \end{array}\right],$$

Applying proper boundary conditions, the following dimensions for the matrices were produced and, therefore, were solved to measure the required force and moment at each eel segment:

(20) A:[3N×(3N+6)]

(21) $$\underline{f}:\left[\left(3N+6\right)\times 1\right]$$

(22) B:[3N×1]

**Figure 5:**
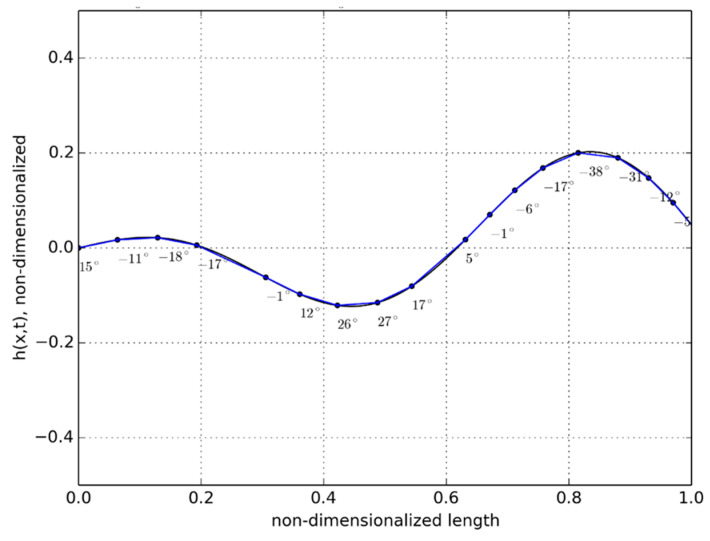
Relative angles at the joints of each segment (t = 2.5 s).

**Figure 6:**
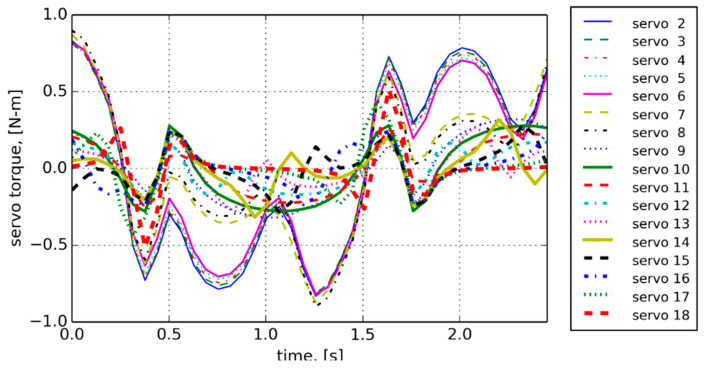
Generated servo torques vs. time.

The generated relative angular rotations for the joint-to-joint junction, shown in Figure 5, required the amount of torque from each servo, as shown in Figure 6. The servo actuators could provide maximum torque of 2.4 Nm (340 ozf.in) with max angular speed of 408 degrees/s, quite sufficient to power the robot for an extended period.

During the entire design process, the anguilliform robot underwent several design iterations to achieve different functionalities and increase the robot’s versatility for a variety of swimming conditions. While the NEELBOT 1.1 operated in the x–y plane (sidewise-undulation) only, NEELBOT 1.2 was further developed to achieve depth control while maintaining the correct anguilliform swimming motion. Three aspects were considered for the depth control: (1) modifying the existing housing rotating from sidewise to up and down, (2) attaching a control surface that would act like a pectoral fin and (3) implementing the concept of depth control mechanism by means of volumetric expansion and retraction. During the investigation, the design of certain robot sections had to be altered to implement the desired functionality. As the sections were altered, the servo torque calculations were completed in order to determine whether or not the existing servos had enough power to perform.

**Figure 7:**
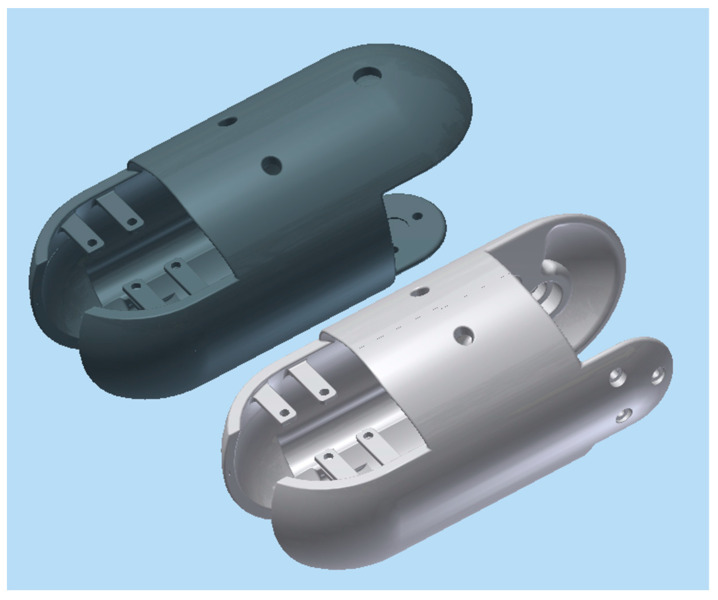
Rotated assembly joint by 90°.

**Figure 8:**
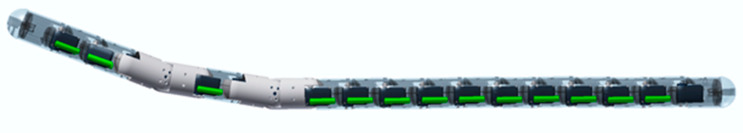
Position of the rotated assembly in NEELBOT 1.2.

To achieve depth control, the first design iteration considered rotating two of the existing servos by 90° to give the robot the ability to redirect thrust in the x–z plane. Two sets of servo housings were also rotated by 90°; the common interface between the third and fourth and the sixth and seventh segments were split and fabricated to match the servo horn position. The fifth servo housing was left unchanged to reduce design variation. Figure 7 shows the rotated interface of the housings and the positions in the housings with the servos are shown in Figure 8.

**Figure 9:**
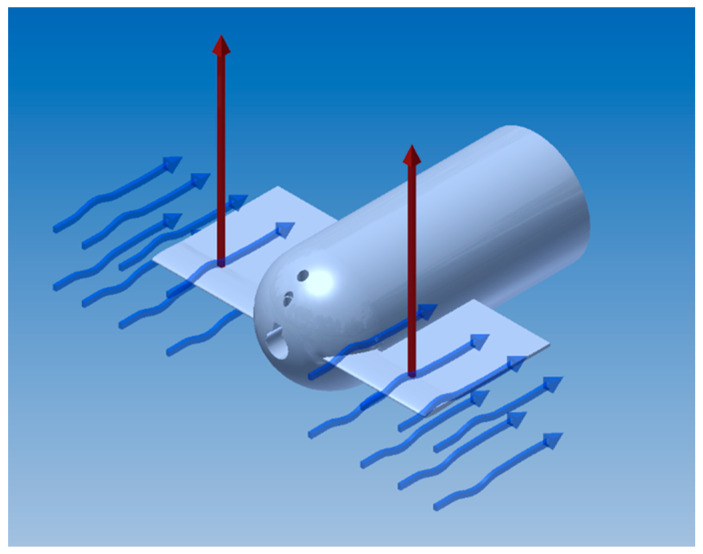
Foil attached to the head piece.

The second iteration consisted of fabricating the head pieces with foils that would act like pectoral fins, as shown in Figure 9. The head piece was fabricated with two positively cambered foils having a length of 73 mm and width of 60 mm. Due to space limitation, the pectoral fins could not be fitted with addition servos and, therefore, did not have the ability to rotate unlike the several different aquatic robot designs that were mentioned earlier.

**Figure 10:**
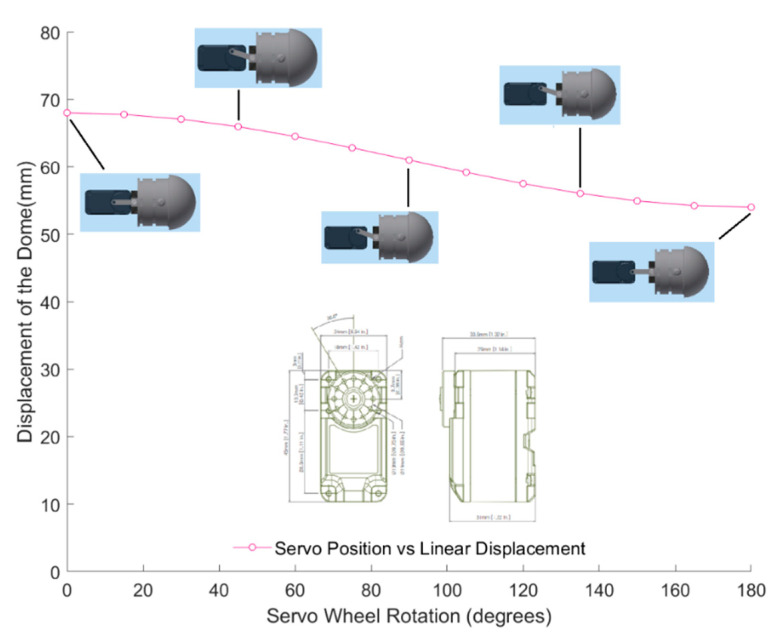
Displacement of the head dome—servo wheel rotated (in degrees).

**Figure 11:**
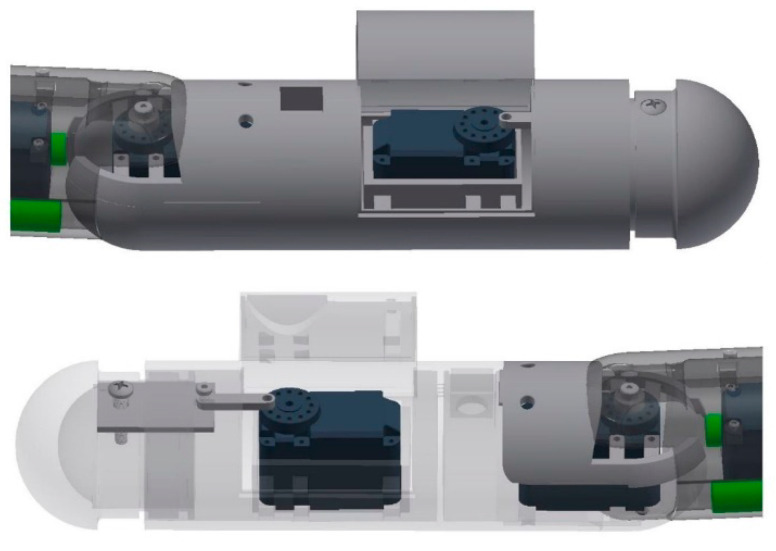
Dome assembly for expansion and retraction showing insert plate, link and servo, front assembly (**top**) and aft assembly (**bottom**).

**Figure 12:**
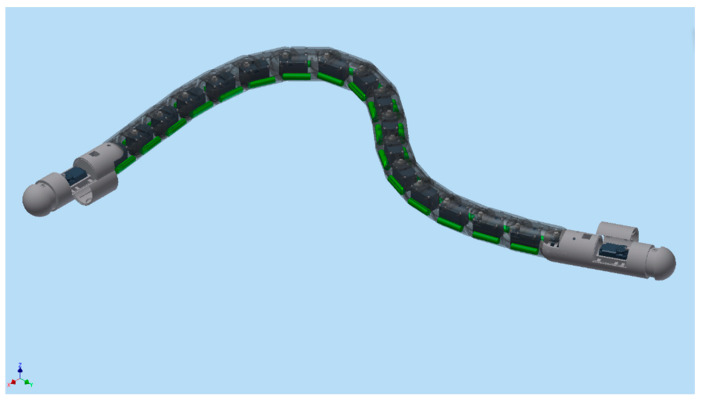
Rendering showing the aft tail and front head pieces with expanding and retracting assembly.

For the third design iteration for depth control, we created a depth control mechanism at the first and last segment. These new segments would have the ability to elongate and shorten to adjust the buoyancy and longitudinal center of buoyancy, as shown in the displacement graph in Figure 10. This would give the robot the ability to control depth and trim. In order to achieve this, the front and the aft segments were lengthened in order to accommodate the linker-plate connected to the servo motor, shown in Figure 11. Due to the rotating motion, it was found that the servo would tend to displace when the linker and the horn wheel were rotated past 90°; therefore, another compartment was fabricated to hold the servo motor in place. During the design experiment, O-rings were placed at the junction of the extending dome and the inner side of the robot cavity to keep the inside of the servo wheel arrangement waterproof. Several design iterations were made to examine the function of the axial movement and were tested in small desk tanks. During desk tank tests, light skin latex balloons were used along with a clamp mechanism in order to avoid repeated skin waterproofing and peeling needed for the final test runs. A complete rendering of the NEELBOT with the modified head and tail assemblies for the actual tow tank test runs is shown in Figure 12.

### 3.2. Stress Analysis

**Figure 13:**
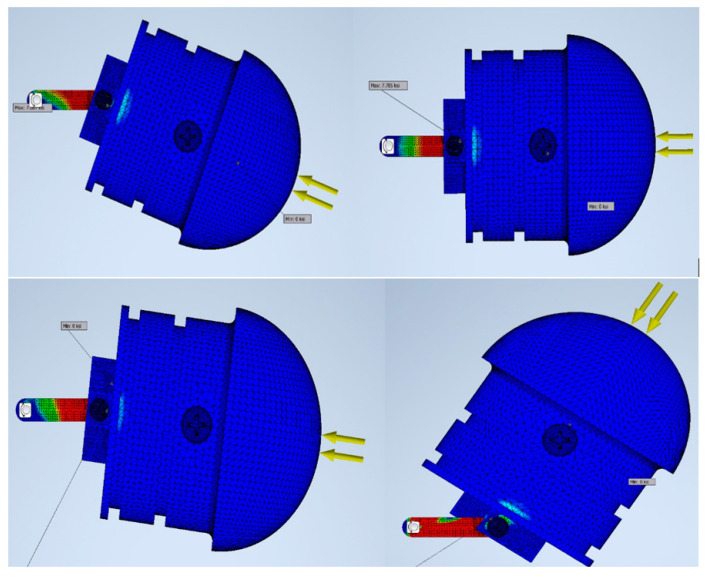
Stress analysis (FEA) used to simulate the dynamic modelling.

**Figure 14:**
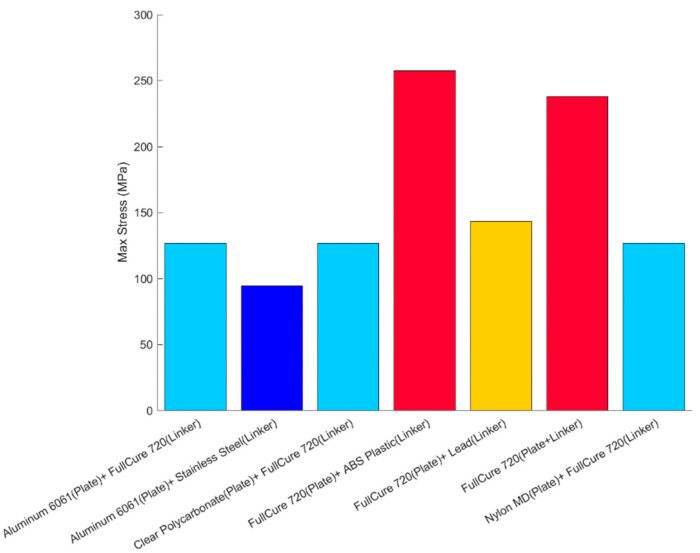
Combination of different materials and maximum stress.

The link and the insert plates were designed after several iterations, since high stress accumulated at the junction where the set screw connected the entire dome assembly, depicted in Figure 13. On the outside of the dome, the primary and largest source of force was due to buoyancy and, thus, increased with the depth of the submersion of the robot. Since the entire eel robot was fabricated with PolyJet FullCure 720, high singular stress points occurred at the servo–collar junction. A series of finite element analyses with different material types was performed, while the dome–linker combination was dynamically rotated at various angles. After several iterations, high stress points were identified, and a small insert plate was used to drive the axial motion of the dome instead of the linker connecting the dome itself. While ABS plastic and complete PolyJet material were damaged after several cycles of axial movement, the combination of aluminum 6061 for the insert plate and stainless steel for the linker performed much better for longer testing periods, as the results indicate in Figure 14.

With the rotated joints at the third–fourth and sixth–seventh segments (Figure 8) and the attached foil next to the head piece (Figure 9), the robotic eel did not perform well in changing height in longer tank tests. In the case of the rotated segments, the hydrostatic pressure at the joints was extremely high and sufficient change in height could not be achieved because of the reduced undulatory motion. With the attached foils, completely functional rotating pectoral fins could not be designed due to space limitation and sufficient lift could not be generated due to the robot’s low forward speed.

**Figure 15:**
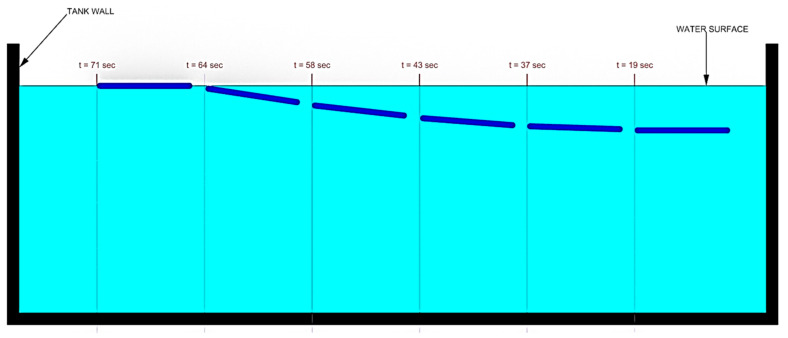
Time lapse of NEELBOT 1.2 swimming while changing depth in the towing tank.

**Figure 16:**
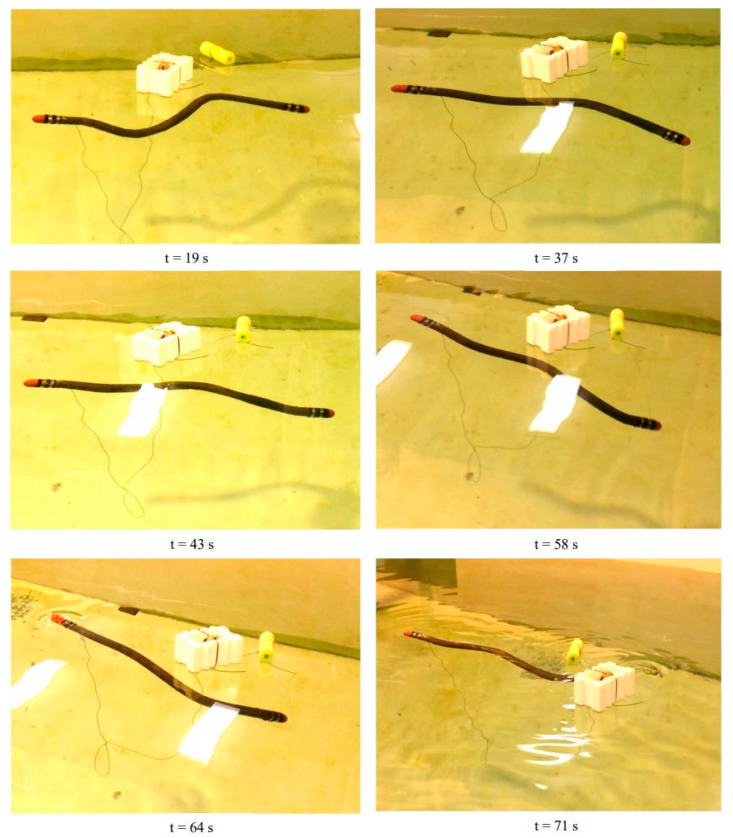
Swimming test of NEELBOT 1.2 with expanding/retracting dome conducted in the towing tank.

For the case of buoyancy control using expanding/retracting head and tail domes, much better results were observed. As depicted in Figure 15, the robot was able to rise to the surface from a depth of approximately 0.7 m in 52 s. The recorded time-lapse series obtained from the actual swimming tests in the tow tank is pictured in Figure 16. While underneath the water surface, the forward and aft domes remained in the contracted position for the first 37 s. The water surface exhibited very minimal disturbance with almost zero swimming-induced wake while the eel thrusted forward. At t = 43 s, the fore and aft dome assembly started to expand and, since the undulatory amplitude for the front section was very small compared to the aft section, the head piece pulled the rest of the eel body towards the water surface. At t = 64 s, the red head dome piece appeared on the water surface at first and after 7 s at t = 71 s, the entire eel body appeared at the water surface. Several tests were repeated consecutively, with the dome contraction pulling the eel down below the water surface level and the expansion bringing up the eel to the surface. The continuous propagative body motion, as shown earlier in Figure 2, was satisfactorily achieved with the combined design change of modified head and tail domes that enabled the robotic eel to change depths in the actual tank tests.

## 4. Conclusions

By utilizing an eel robot that was originally designed to produce an ideal swimming motion as proposed by Vorus and Taravella [12], the authors were able to design, implement and test a mechanism that would control the depth of the robot. Three separate methods were investigated, with the buoyancy control mechanism showing promising results.

The depth control mechanism with the rotated segments at the two distinctive segment positions did not provide adequate results, due to the inability of the robot to generate enough redirected thrust utilizing the remaining segments aft of the servo rotation. The depth control mechanism with control surfaces (foils) attached to the head piece did not achieve satisfactory results either, due to the robot’s design for low speed and inability to produce enough hydrodynamic lift at the foils. These results are similar to the findings of Guo et al. [14], Zhang et al. [16] and Yu et al. [13], in which they found that a large propulsion system driving pectoral fins with large surface area was needed for high energy utilization. Through our design experiment of the robotic eel, it was not realizable because of the space limitations in the slender body design needed to achieve a very high hydrodynamic efficiency. 

The third design selection of controlling the buoyancy by axially moving the front and aft domes is similar to the design by recent researchers [19,20,22], but implemented inside of the robotic eel body and tested in the tow tank setting. During the tests, the design with the expanding head/tail provided excellent results for depth and trim control. The robot was easily able to surface and dive while self-adjusting its buoyancy and center of buoyancy. Throughout the depth change experiment, the anguilliform eel robot maintained its ability to swim with the ideal undulatory motion.

## Figures and Tables

**Table 1:** Robot design parameters.

Parameter	NEELBOT w/o Depth Control	NEELBOT w/Extending Hemispheric End Domes
U_0_ (m/s)	0.25	0.25
Advance ratio	0.7	0.7
No. of segments	19	19
Total length (m)	1.30	1.35
Nominal length (m)	0.972	0.920
Max torque (Nm)	0.678	0.809

## Data Availability

Not applicable.
